# Impact of dietary nano-scale zinc oxide supplementation on growth performance, jejunum morphology, and intestinal microbiota in broilers

**DOI:** 10.3389/fmicb.2026.1886279

**Published:** 2026-07-09

**Authors:** Feng He, Qiushi Wei, Xinyue Zou, Xizhuo Yang, Shan Fang, Zeliang Chen, Caimei Shao, Mingxiao Ma, Desheng Li

**Affiliations:** 1 College of Husbandry and Veterinary Medicine, Jinzhou Medical University, Jinzhou, China; 2 Liaoning Provincial Key Laboratory of Animal Product Quality and Safety, Jinzhou Medical University, Jinzhou, China; 3 Collaborative Innovation Center for Prevention and Control of Zoonoses, Jinzhou Medical University, Jinzhou, China; 4 Wellhope Food Co., Ltd., Shenyang, China

**Keywords:** broilers, growth performance, gut microbiota, intestinal morphology, Nano-ZnO

## Abstract

**Objectives:**

Given the widespread adoption of nanotechnology, this study aimed to investigate the utilization of zinc nanoparticles as a dietary supplement for broiler chickens.

**Methods:**

A total of 480 day-old Arbor Acres Plus broilers (mixed-sex) were used to evaluate the effects of dietary zinc oxide nanoparticles (Nano-ZnO) on growth performance, jejunum morphology, blood parameters and cecal microbiota. All poultry were randomly divided into 4 groups according to initial body weight. Each group had six repeating cages with 20 chickens in each cage. The experiment lasted for 42 days. Dietary treatment was based on the base diet and supplemented with 25, 50, 100 mg/kg Nano-ZnO.

**Results:**

Our results showed that Nano-ZnO improved the growth performance and jejunal villus morphology of broilers (*p* < 0.05). Blood parameter analysis revealed that 25 mg/kg Nano-ZnO significantly increased serum levels of albumin (ALB), total protein (TP), and globulin (GLB) (*p* < 0.05). Moreover, compared with the Con group, the 25 mg/kg Nano-ZnO group exhibited higher relative abundances of *Bacteroides*, *Parasutterella*, *UCG-055*, and *Parabacteroides* at the genus level in the cecal microbiota. Additionally, Nano-ZnO increased the abundance of *Lactobacillus* in fecal microorganisms (*p* < 0.05).

**Conclusion:**

In summary, we conclude that dietary supplementation with 25 mg/kg of Nano-ZnO is an effective approach for enhancing the growth performance of broiler chicks. It achieves this by optimizing intestinal morphology and cecal microorganisms.

## Introduction

White-feathered broiler chickens are extensively reared in intensive farming systems worldwide. These birds are characterized by rapid growth, high metabolic activity, short reproductive cycles, and strong nutrient utilization efficiency (Luo et al., 2024). However, under conventional high-density housing conditions, broilers frequently experience intestinal dysfunction, reduced nutrient digestibility, and impaired immune function throughout the rearing period. In the past, the widespread use of antibiotics was the primary approach to mitigate these issues. Nevertheless, the potential spread of antibiotic-resistant pathogenic strains into the environment, along with the risk of human infection through the food chain, poses serious public health concerns (Mohamed et al., 2022). The restriction of antibiotic use as growth promoters in livestock production has therefore prompted the evaluation and development of novel alternatives to promote growth (Tang et al., 2017). Among these alternatives, nano-scale zinc oxide (Nano-ZnO) has attracted extensive attention due to its growth-promoting and antibacterial properties, as well as its regulatory effects on animal immune and reproductive functions (Alian et al., 2023).

Zn acts as an essential cofactor for numerous enzymZinc (Zn) serves as an essential micronutrient in poultry production, fulfilling pivotal roles in immunological homeostasis, reproductive endocrinology modulation, tissue regeneration, and nucleic acid (RNA/DNA) biosynthesis (Lilburn and McIntyre, 2024). Conventional zinc supplementation in animal feed predominantly relies on inorganic zinc compounds, notably zinc oxide (ZnO) and zinc sulfate (Luo et al., 2024). However, prolonged supra-nutritional administration of these zinc sources may result in resource inefficiency, antagonistic interactions with concurrent mineral absorption, and potential zinc toxicity (Ming et al., 2024). In contrast, Nano-ZnO characterized by its quasi-spherical morphology and particle diameters of 1–100 nm (Madl et al., 2014), demonstrates enhanced biophysical properties. Owing to its reduced particle size and elevated surface-area-to-volume ratio, Nano-ZnO exhibits superior gastrointestinal permeability and bioavailability compared to conventional zinc formulations (El-Gindy et al., 2023). In addition, Nano-ZnO exhibits potent desiccant, anti-inflammatory, and antibacterial properties (Hashemi et al., 2023). Its bactericidal activity is attributed to nanoparticle interactions with bacterial membranes, increasing permeability and inducing oxidative stress, ultimately leading to microbial cell death (Fragou et al., 2023; Ahmad et al., 2020). Beyond its antimicrobial effects, Nano-ZnO modulates gut microbiota composition, promoting beneficial bacterial populations while suppressing pathogenic strains (Yang et al., 2024). This is particularly significant in animal production, where gut microbial ecology directly influences nutrient utilization and growth performance. Recent studies suggest that dietary Nano-ZnO supplementation enhances broiler immunity and antioxidant capacity while selectively enriching short-chain fatty acid (SCFA)-producing bacteria (e.g., Lactobacillus, Bifidobacterium) and inhibiting harmful taxa (e.g., Clostridia, Bacteroides) (Qu et al., 2023). Such modulation may improve intestinal health and metabolizable energy efficiency, though further research is needed to elucidate its full mechanistic role in poultry.

In light of these encouraging findings, we initiated a study to investigate the impact of dietary supplementation with Nano-ZnO on growth performance, blood parameters, gut microbiota, and excreta bacterial populations in broiler chicks. Our hypothesis is that this intervention will improve growth performance and blood parameters by enhancing gut microbes and small intestine morphology. This study aims to address knowledge gaps regarding the potential benefits of Nano-ZnO in poultry nutrition and production, ultimately contributing to the development of more efficient and sustainable poultry farming practices.

## Materials and methods

### Preparation of nano zinc oxide

Nano-ZnO is provided by the School of Pharmacy of Jinzhou Medical University. Its average particle diameter is 5 nanometers. The nano-zinc oxide appears spherical under the microscope and contains no impurities. The preparation method is as follows: First, 10.0 mmol of NaOH was dissolved in 100.0 mL of absolute ethanol at 50 °C under continuous stirring for 20 min. At the same time, 10.0 mmol of zinc acetate dihydrate was dissolved in 100.0 mL of ethanol at 65 °C with stirring for 20 min to form a zinc precursor solution. After the solution was cooled to 50 °C, PEG200 was added to the zinc precursor to achieve final concentrations of 5, 10, 20, 40, and 80 μM, respectively, followed by stirring for 10 min. Subsequently, the NaOH solution was rapidly added to the PEG-containing zinc solution under vigorous stirring, and the mixture was stirred for an additional 30 s. The reaction was then terminated by adding 200 mL of distilled water. The resulting mixture was centrifuged at 8,000 rpm for 10 min. The supernatant was discarded, and the precipitate was washed twice with ethanol to remove residual reactants. The purified Nano-ZnO quantum dots were dried under vacuum at 60 °C for 48 h (Niu et al., 2025).

### Experimental design

In this experiment, a single - factor experimental design was employed. All birds are provided by the Animal Experiment Center of Jinzhou Medical University. A total of 480 one-day-old Arbor Acres Plus broilers with body weights close to the healthy range were carefully selected (mixed-sex). These broilers were then randomly allocated into 4 treatment groups. Each group consisted of 6 replicate cages, and each replicate cage housed 20 chickens. The control group (Con) was provided with a basal diet (without additional Zn). Experimental group 1 (ZnO 25) received the basal diet supplemented with 25 mg/kg Nano-ZnO. Experimental group 2 (ZnO 50) was fed the basal diet with an addition of 50 mg/kg Nano-ZnO. Experimental group 3 (ZnO 100) was given the basal diet fortified with 100 mg/kg Nano-ZnO. The formal phase of the experiment was set to last for 42 days. The experimental chickens were housed in a three - layer broiler cage system. The barn was a closed environment equipped with a temperature control device. The initial temperature was set at 33 °C and gradually decreased by 3 °C per week until it reached 22 °C. The chickens had ad libitum access to feed and water, and were vaccinated on schedule. The condition of the experimental chickens was monitored daily. Regarding the immunization procedure, 7-day-old chickens were vaccinated with a triple inactivated vaccine against Newcastle disease and avian influenza virus. At 21 days old, the chickens were immunized via Newcastle disease drinking-water vaccination. The feeding management and poultry house health protection of the experimental chickens were implemented in accordance with the feeding guidelines for white - feathered broilers. The basal diet and its nutrient levels were formulated based on the nutritional requirements of NRC (1994) and NY/T 903-2004 for broiler chickens, as presented in Table 1.

**Table 1:** Composition and nutritional level of basal diet (air-dried basis).

Ingredients, %	1–21日龄	22–42日龄
Corn	59.05	57.75
Soybean oil	2.00	6.00
Corn gluten meal (60%)	4.00	4.00
Bean meal (45%)	30.5	28.50
Limestone flour	1.40	1.00
Calcium bicarbonate	1.30	1.00
Nacl	0.25	0.25
Premix^1^	1.50	1.50
Total	100	100
Analyzed composition, %		
Metabolizable energy, MJ/kg	12.80	13.89
Crude protein/%	23.00	21.00
Ca/%	0.90	0.75
Total phosphorus/%	0.60	0.50
Lysine/%	1.415	1.308
Methionine/%	0.563	0.456
Methionine + cystine/%	0.971	0.881

1 Provided per kg of complete diet: VA 10,000 IU, VD3 4,000 IU, VE 40 IU, VK 4 mg, VB_1_ 5 mg, VB_2_ 8 mg, niacin 65 mg, pantothenic acid 20 mg, VB_6_ 5 mg, biotin 0.5 mg, folic acid 2 mg, VB_12_ 0.02 mg, copper 9 mg, iron 85 mg, manganese 0.6 mg, iodine 0.6 mg.

### Growth performance

During the experimental period, daily feed intake was recorded for all replicate cages. Body weights of broilers were measured on days 1, 21, and 42 post-hatching, with zootechnical parameters including average daily gain (ADG), average daily feed intake (ADFI), and feed to gain ratio (F/G) being calculated for three distinct growth phases: starter (1–21 days), grower (21–42 days), and overall trial period (1–42 days).

### Hematology parameters analysis

Eighteen broilers per experimental group (three birds per replicate cage) were randomly selected for blood collection. On the morning of day 42, venous blood samples were collected from the wing vein using plain vacuum tubes. Following 15-min coagulation at room temperature, samples were centrifuged at 3,000 × g for 15 min at 4 °C to obtain serum. The levels of albumin (ALB), total protein (TP), globulin (GLB), albumin-to-globulin ratio (A/G), aspartate aminotransferase (AST) and alanine aminotransferase (ALT) in the blood were measured using a fully automatic blood biochemical analyzer (Trilogy, DREW Company, United States).

### Intestinal morphology observation

These broilers were euthanized by anesthesia via wing vein injection of pentobarbital sodium at 50 mg/kg of body weight, in order to avoid the violent struggle of broilers during bleeding that might affect the intestinal morphology. Jejunal segments were dehydrated using a graded series of xylene and ethanol after fixation in paraformaldehyde solution for 24 h. After that, they were embedded in paraffin, and then specimens were sliced into 5-micron-thick sections and dyed with hematoxylin and eosin for morphological evaluation. The intestinal morphology images were obtained using a virtual microscope (Nikon 80i, Tokyo, Japan). Intestinal villus height (VH), which means from the tip of the villus to the villus-crypt junction, and crypt depth (CD) were measured from the crypt opening to the crypt base (Woyengo et al., 2011). All data were measured by Image-Pro Plus software. Finally, to calculate the ratio of villus height to crypt was defined as VH/CD. Fifteen intact villi and adjacent crypts were randomly selected from each section for the evaluation of jejunal morphology.

### DNA extraction and 16S rRNA sequencing

On the morning of day 42, six broilers from each group were selected for sample collection. The birds were euthanized after being anesthetized by an intravenous injection of sodium pentobarbital (50 mg/kg body weight) via the wing vein. The cecum was ligated at both ends with a sterile silk suture and then transferred into a laminar flow hood. Within the hood, the cecal contents were carefully extruded by gentle squeezing and collected into a sterile cryovial. Total DNA was extracted from cecal digesta samples using a rapid DNA SPIN extraction kit. PCR products were separated and recovered by 2% agarose gel electrophoresis, followed by quantification with a Quant-iT™ PicoGreen® dsDNA detection kit (Calbiochem). Sequencing was performed by Shanghai Personal Biotechnology Co., Ltd. (Shanghai, China). Raw sequences were processed using FLASH software (v1.2.7) for merging and quality filtering. Sequences were then clustered into operational taxonomic units (OTUs) at a 97% similarity threshold. The number of OTUs per group was counted and visualized using Venn diagrams in R software (version 2.15.3). Principal component analysis (PCA) was conducted and plotted with R software. Linear discriminant analysis effect size (LEfSe) was performed using the LEfSe tool, with an LDA score threshold set to 3.0 to identify differentially abundant taxa between groups. For species showing significant intergroup differences, a two-sample t-test was applied in R, and corresponding visualizations were generated.

### Excreta bacteria

The test period was 42 days old. Fresh feces of the test chickens were collected and excreta samples were collected from each replicate cage. In order to determine the number of viable bacteria in the excreta samples, 1 g sample was mixed with 9 mL sterile peptone water for 1 min after mixing the feces samples of each replicate. The prepared mixture was diluted 10 times continuously and coated on a specific agar plate. *Lactobacilli* MRS agar, MacConkeyagar, and *Salmonella*-Shigella agar (Difco Laboratories, Detroit, MI) plates were employed to isolate *Lactobacillus*, *E. coli*, and *Salmonella*. Subsequently, the lactobacilli agar plates were incubated for 24 h at 37 ° C under anaerobic. The colonies of each bacterium were counted and expressed in logarithms of colony-forming units per gram (Maharjan et al., 2019).

### Statistical analysis

The results are expressed as mean ± standard deviation (mean ± S. D.). Statistical analysis passed the least squares analysis of variance, following a general linear model, and the statistical data obeyed a normal distribution. Statistical analysis was performed using Prism software (GraphPad Software, San Diego, CA, United States), One-way analysis of variance (ANOVA) and Tukey’s *post-hoc* test were used for the statistical analysis of more than two groups. *p* < 0.05 were considered statistically significant.

## Results

### Effect of nano-scale zinc oxide on growth performance of broilers

As shown in Table 2, compared with Con, the ADG of ZnO 25 group at 1–21 and 1–42 days of age was significantly increased (*p* < 0.05). The ADG of 1–21 and 1–42 days old in ZnO 50 and ZnO 100 groups increased but the difference was not significant (*p* > 0.05). The F/G of 1–42 days of age in the ZnO 25 group was significantly decreased (*p* < 0.05).

**Table 2:** Effects of dietary supplementation of Nano-ZnO on the growth performance of broiler chicks.

Items	Con	ZnO 25	ZnO 50	ZnO 100
ADG (g/d)				
1–21	44.26 ± 0.40^b^	44.81 ± 0.35^a^	44.67 ± 0.33^ab^	44.45 ± 0.40^ab^
22–42	88.55 ± 0.82	90.99 ± 2.16	89.63 ± 1.33	89.31 ± 3.37
1–42	66.40 ± 0.33^b^	67.90 ± 0.95^a^	67.15 ± 0.65^ab^	66.88 ± 1.59^ab^
ADFI (g/d)				
1–21	51.15 ± 0.49	51.48 ± 0.13	51.36 ± 0.13	51.39 ± 0.23
22–42	145.26 ± 0.41	145.34 ± 0.24	145.29 ± 0.18	145.15 ± 0.12
1–42	97.74 ± 0.74	97.39 ± 1.13	97.68 ± 0.23	97.64 ± 0.77
F/G (g/g)				
1–21	1.16 ± 0.01	1.15 ± 0.01	1.15 ± 0.01	1.16 ± 0.01
22–42	1.64 ± 0.02	1.60 ± 0.04	1.62 ± 0.02	1.63 ± 0.06
1–42	1.47 ± 0.01^a^	1.43 ± 0.01^b^	1.45 ± 0.02^ab^	1.46 ± 0.04^ab^

ADG, average daily gain; FI, feed intake; SD, standard deviation.

a,b Means in the same row with different superscript indicates a significant difference (*p* < 0.05), while no superscript indicates no significant difference among groups (*p* > 0.05).

### Effect of Nano-ZnO on Hematology parameters of broilers

As shown in Table 3, compared with the Con group, the levels of ALB, TP, and GLB in the ZnO 25 group were significantly increased (*p* < 0.05), while the A/G was significantly decreased (*p* < 0.05). No significant differences in the levels of AST and ALT were observed among the ZnO 25, ZnO 50, and ZnO 100 groups (*p* > 0.05).

**Table 3:** Effects of dietary supplementation of Nano-ZnO on the hematology parameters of broiler chicks.

Items	Con	ZnO 25	ZnO 50	ZnO 100
ALB	11.92 ± 0.31^b^	12.60 ± 0.24^a^	12.10 ± 0.31^b^	12.20 ± 0.46^ab^
TP	31.05 ± 0.60^b^	33.50 ± 1.07^a^	32.07 ± 0.41^ab^	33.25 ± 3.00^ab^
GLB	18.85 ± 0.45^c^	21.52 ± 0.50^a^	20.82 ± 1.11^ab^	19.93 ± 0.51^b^
A/G	0.63 ± 0.03^a^	0.59 ± 0.01^b^	0.58 ± 0.04^b^	0.61 ± 0.02^ab^
AST	316.17 ± 34.67	298.33 ± 31.23	303.33 ± 39.99	302.5 ± 25.30
ALT	9.67 ± 1.25	9.17 ± 1.95	9.83 ± 1.57	9.75 ± 2.03

ALB, albumin; TP, total protein; GLB, globulin; A/G, albumin to globulin ratio; AST, aspartate aminotransferase; ALT, alanine aminotransferase; SD, standard deviation.

a,b Means in the same row with different superscript indicates a significant difference (*p* < 0.05), while no superscript indicates no significant difference among groups (*p* > 0.05).

### Effects of dietary Nano-ZnO on intestinal morphology of jejunum in broilers

As shown in Table 4, compared with Con group, the villus length and V/C of jejunum in ZnO 25 group were significantly increased (*p* < 0.05). The villus length and V/C of jejunum in ZnO 50 and ZnO 100 groups were increased but the differences were not significant (*p* > 0.05). There was no significant difference in jejunal crypt depth between ZnO 25 group, ZnO 50 group and ZnO 100 group (*p* > 0.05).

**Table 4:** Effects of dietary Nano-ZnO on intestinal morphology of jejunum in broilers.

Items	Con	ZnO 25	ZnO 50	ZnO 100
Villus length (μm)	1492.77 ± 33.21^b^	1530.37 ± 15.27^a^	1508.15 ± 25.34^ab^	1497.54 ± 25.59^ab^
Crypt depth (μm)	205.53 ± 8.95	189.82 ± 3.87	195.67 ± 20.45	203.73 ± 7.76
V/C	7.36 ± 0.46^b^	8.07 ± 0.19^a^	7.79 ± 0.81^ab^	7.36 ± 0.32^ab^

SD, standard deviation.

a,b Means in the same row with different superscript indicates a significant difference (*p* < 0.05), while no superscript indicates no significant difference among groups (*p* > 0.05).

### Effects of dietary supplementation of Nano-ZnO on the excreta bacteria of broiler chicks

As shown in Table 5, compared with Con group, the number of *Lactobacillus* in excreta of ZnO 25, ZnO 50 and ZnO 100 groups at 42 days of age was significantly increased (*p* < 0.05), while the number of *Escherichia coli* and *salmonella* in excreta of ZnO 25, ZnO 50 and ZnO 100 groups at 42 days of age had no significant effects (*p* > 0.05).

**Table 5:** Effects of dietary supplementation of Nano-ZnO on the excreta bacteria of broiler chicks.

Items	Con	ZnO 25	ZnO 50	ZnO 100
*Escherichia coli* (log_10_ CFU/g)	5.40 ± 0.85	4.69 ± 0.90	4.99 ± 0.95	5.26 ± 0.75
*Salmonella* (log_10_ CFU/g)	2.52 ± 0.21	2.17 ± 0.63	2.46 ± 0.50	2.34 ± 0.53
*Lactobacillus* (log_10_ CFU/g)	7.88 ± 0.73^b^	8.98 ± 0.15^a^	8.66 ± 0.30^a^	8.66 ± 0.46^a^

SD, standard deviation.

a,b Means in the same row with different superscript indicates a significant difference (*p* < 0.05), while no superscript indicates no significant difference among groups (*p* > 0.05).

### Microbe composition of cecum

**Figure 1:**
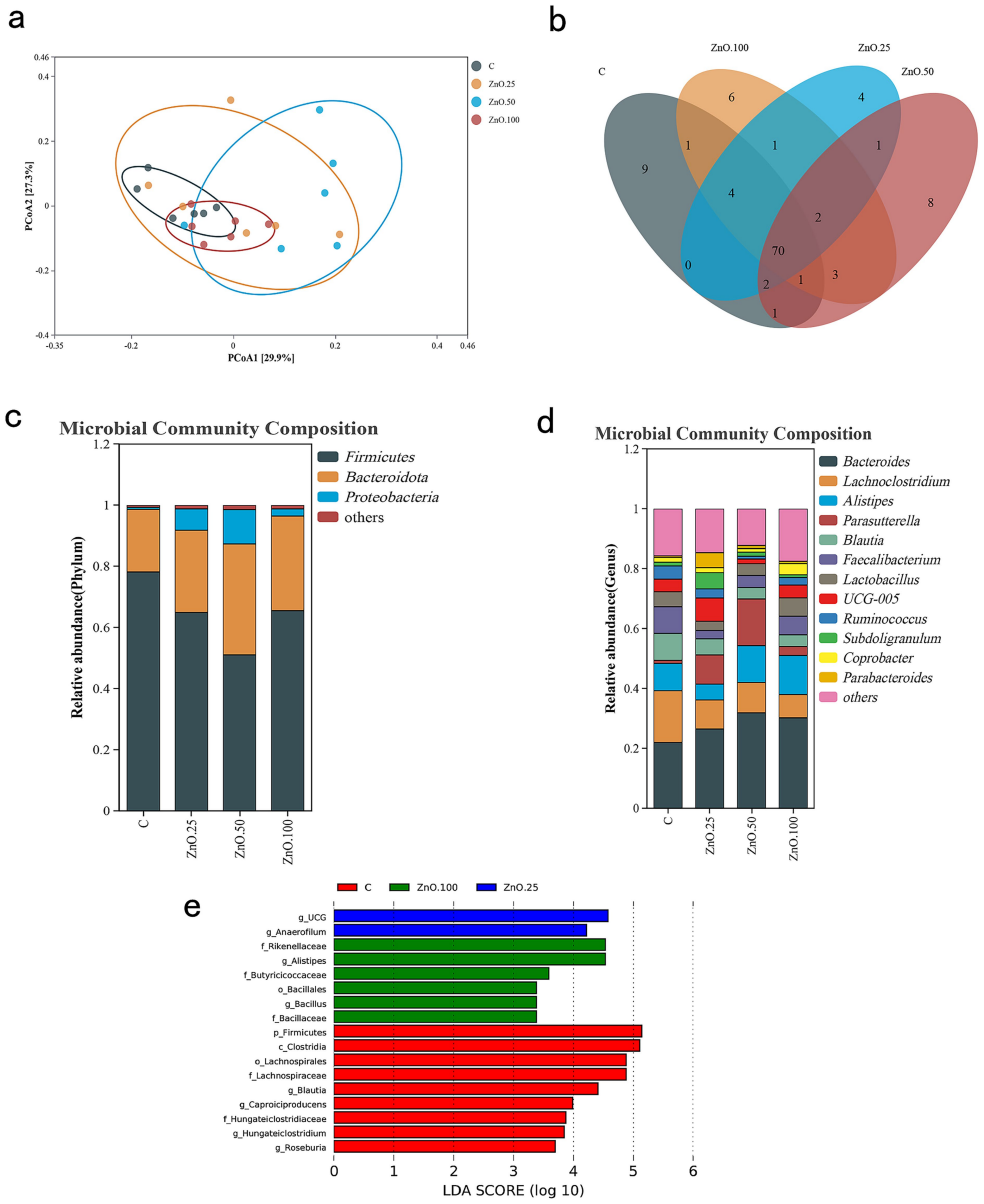
Effect of nano-scale zinc oxide on intestinal flora of broilers. Cecal PCoA between groups **(a)**. Venn diagram of cecal microbiota **(b)** amplicon sequence variation (ASV). At the phylum level **(c)**, the relative abundance of cecal bacteria between groups. At the genus level **(d)**, the relative abundance of cecal bacteria. Linear discriminant analysis (LDA) was used to compare the biomarker types **(e)** of cecal microbiota between groups.

In order to explore the effect of Nano-ZnO on intestinal microorganisms, we detected cecal microorganisms. As shown in Figure 1a, there was no obvious separation of microorganisms among the groups. The Venn diagram of the effect of Nano-ZnO on the number of cecal microorganisms in white-feathered broilers is shown in Figure 1b. The numbers with overlapping parts in the Venn diagram indicate the number of OTUs shared by different groups, and the numbers without overlapping parts indicate that the group has a unique number of OTUs. There were 70 OTUs in the four treatment groups, including 9 unique OTUs in the Con, 4 unique OTUs in the ZnO 25 group, 8 unique OTUs in the ZnO 50 group, and 6 unique OTUs in the ZnO 100 group. In this experiment, the relative abundance of phylum was greater than 1% as the criterion for determining the dominant phylum. The effect of Nano-ZnO on the composition of cecal microbial phylum in white feather broilers is shown in Figure 1c. Compared with the Con group, the relative content of *Bacteroides* and *Proteobacteria* in the cecal contents of the ZnO 25, ZnO 50 and ZnO 100 groups increased, and the relative content of *Firmicutes* decreased. At the genus level (Figure 1d), compared with the Con group, the relative contents of *Bacteroides*, *Parasutterella*, *UCG*-*055* and *Parabacteroides* in the cecal microbial genus level of the experimental ZnO 25 group increased, and the relative contents of *Lachnoclostridium*, *Blautia* and *Faecalibacterium* decreased. The LEfSe analysis results of Nano-ZnO on cecal microorganisms of white feather broilers are shown in Figure 1e. LEfSe analysis enables comparison of differences between multiple groups and internal subgroup comparisons after group comparisons in order to search for species (Biomaker) with significant differences in abundance. The significance of species enrichment among different groups is shown by the length of the bar chart. The results showed that the marker species of ZnO 25 group were UCG-055 and *anaerobicfilum*. The marker species of ZnO100 group are *Alistipes* and *Bacillus*. In the control group, the marker species were *Blautia*, *Caproiciproducens*, *Hungateiclostridium* and *Roseburia*.

## Discussion

The growth performance of white - feathered broilers serves as the most intuitive indicator for assessing the impact of incorporating Nano-ZnO into their diet. Numerous researchers have previously supplemented broiler diets with zinc in either inorganic or organic forms (Naila et al., 2019), and they have witnessed enhancements in the growth performance of broilers. Compared with traditional zinc preparations, Nano-ZnO possesses a larger contact area, which enables it to strengthen the interaction with biofilms and expedite the cellular absorption of zinc (Agarwal and Shanmugam, 2020). ADG, ADFI, and F/G are crucial indicators for gaging economic benefits in broiler production. These parameters can directly mirror the growth performance of animals and the expected economic returns. Currently, in the pursuit of environmentally and economically sustainable development in broiler production, improving feed utilization efficiency has emerged as a central focus (Siegel, 2014). In our study, the addition of 25 mg/kg Nano-ZnO led to an increase in ADG, ADFI, and an improvement in the F/G ratio during the periods of 1–21 days, 22–42 days, and the overall 1–42 day trial. Previous studies have demonstrated that an appropriate level of Nano-ZnO (20 mg/kg diet) can effectively enhance the weight gain of white - feathered broilers (Zhao et al., 2014), which is in line with the findings of our research The inclusion of Nano-ZnO in the diet can fulfill the poultry’s requirement for zinc and maintain the balance of trace elements in their bodies (Dosoky et al., 2022). It has been reported that the improvement of feed utilization may be due to the fact that zinc promotes the improvement of feed utilization by participating in the metabolism of carbohydrates, lipids and proteins (Siegel, 2014). In addition, our data revealed that high concentrations of Nano-ZnO did not exert beneficial effects on growth performance but rather induced certain negative effects. This may be attributed to the fact that when the concentration of Nano-ZnO increases to 50 or 100 mg/kg, the complex intestinal environment (e.g., pH, ionic strength) promotes the aggregation of these high-surface-energy nanoparticles into larger particles. Once agglomerated, they lose their Nano-scale advantages and behave similarly to conventional bulk ZnO, leading to a sharp decline in bioavailability. In summary, adding 25 mg/kg of Nano-ZnO to the diet has the effect of improving the growth performance of broilers.

Serum biochemical indices are excellent indicators of the immune system’s functionality. ALB and GLB, as blood - borne proteins, are of paramount importance to the animal organism. They are responsible for transporting metabolic products and nutrients throughout the body and maintaining the body’s colloid osmotic pressure (Su et al., 2023). GLB, also referred to as immunoglobulin, is a blood protein synthesized by the liver and plays a pivotal role in the human immune system (Li et al., 2019; Igwe et al., 2022). A deficiency of zinc in livestock and poultry leads to a decrease in the content of GLB and ALB, which disrupts the body’s protein metabolism and ultimately results in a decline in production performance (Liu et al., 2023). The incorporation of Nano-ZnO into the diet can safely and effectively augment the body’s zinc content, elevate the levels of ALB and GLB, and thereby enhance the body’s humoral immune response (Ma et al., 2023). In our study, Nano-ZnO supplementation increased the serum levels of ALB and GLB, suggesting that Nano-ZnO may enhance the immunity of broilers. Moreover, we observed an increase in the blood total protein level following Nano-ZnO supplementation. Total protein comprises albumin and globulin, with globulin (e.g., immunoglobulin IgG, IgA, IgM) being an integral component of the immune system (Germitsch et al., 2025). The rise in total protein content indirectly reflects an improvement in the body’s immune capacity. AST and ALT are crucial enzymes in livestock. The results of their determination can accurately reflect the extent of liver injury (Ramesh et al., 2012). Under normal physiological conditions, the levels of AST and ALT remain relatively stable. However, when the liver suffers pathological damage, the levels of these two enzymes increase significantly (Tang et al., 2012). High-dose zinc oxide supplementation can exacerbate liver damage, and the levels of AST and ALT can clearly indicate the extent of Nano-ZnO induced hepatocyte damage (Cho et al., 2015). Our study demonstrated that different concentrations of Nano-ZnO had no impact on the blood levels of AST and ALT, indicating that Nano-ZnO did not cause liver damage in broilers, which validates the safety of the doses used in this study. In conclusion, the addition of Nano-ZnO to the feed did not harm the broilers’ livers and may enhance their humoral immunity.

The intestinal barrier of chickens, primarily constituted by intestinal villi, is regulated by a multitude of factors, including physiological functions and pathological stimuli. A well - functioning intestinal barrier is pivotal for enhancing the body’s growth performance (Awad et al., 2017). The jejunum serves as the primary site for nutrient digestion and absorption in chickens. Its absorptive capacity is largely dependent on the intestinal villi, and there is a positive correlation between the length of these villi and the efficiency of nutrient absorption (El Aidy et al., 2015). When the intestinal villi come into contact with fine chyme particles, a larger contact surface area allows the intestine to augment both the quantity and rate of nutrient absorption. The barrier function of the small intestine is sustained by the remarkable self - renewal rate of epithelial cells. This renewal process is predominantly attributed to the proliferation and differentiation within the crypts. The crypt represents a distinctive biological system, mainly composed of intestinal stem cells and Paneth cells (De Grande et al., 2020). Even though bacteria can penetrate the small - intestine mucus, epithelial cells continuously secrete mucus and antibacterial factors. The goblet cells within the small intestine crypts secrete a substantial amount of mucin particles, which are rapidly expelled to eliminate bacteria from the crypts and maintain a sterile environment within them (Dolan et al., 2022). Previous research by Long et al. indicated that the addition of Nano-ZnO could effectively reduce the depth of ileal crypts in weaned piglets, exert a favorable anti - inflammatory and astringent effect on intestinal morphology, and effectively prevent diarrhea (Long et al., 2017). Studies have shown that the addition of Nano-ZnO may enhance the self-regeneration and reproductive capabilities of intestinal stem cells, which is beneficial for the development of the duodenum and ileum (Wang et al., 2017). Our findings indicate that dietary supplementation with 25 mg/kg of Nano-ZnO can elevate the villus height-to-crypt depth ratio in the jejunum. This elevation promotes more efficient absorption of nutrients in the intestinal tract. The changes of intestinal morphology also showed that Nano-ZnO improved intestinal immunity. As a consequence of these beneficial effects, there is an increase in the body weight of the livestock.

The intestinal flora of broilers is of paramount significance in safeguarding host health, maintaining intestinal barrier function, facilitating nutrient absorption, and enhancing production performance (Jian et al., 2021). A higher diversity of the intestinal flora is associated with greater stability. Previous studies have suggested that Nano-ZnO exhibits antibacterial and bactericidal properties, potentially leading to a reduction in the diversity of the intestinal flora. However, in our investigation, different concentrations of Nano-ZnO did not impact the quantity of the intestinal flora. The cecum of poultry is replete with a vast number of microorganisms, serving as an ideal environment for the robust growth and reproduction of various microbial species. The two cecal diverticula can augment the microbial abundance and extend toward the rectum (Sergeant et al., 2014). The intestinal bacteria of poultry are mainly *Bacteroidetes*, *Firmicutes* and *Proteobacteria*, accounting for more than 90% of the total bacterial community (Huang et al., 2018), which is consistent with our results. In addition, in our study, Nano-ZnO increased the relative content of *Bacteroides* and *Proteobacteria*, and reduced the relative content of *Firmicutes*. At the genus level, the relative contents of *Bacteroides*, *Parasutterella*, *UCG*-*055* and *Parabacteroides* in cecum were increased in ZnO 25 group. And the relative content of Gram-positive bacteria *Lachnoclostridium*, *Blautia*, and *Faecalibacterium* were reduced. *Bacteroides* are widely found in the intestinal tract of animals, and their main function is to fermentate polysaccharides in the intestinal tract and produce short-chain fatty acids (acetate and propionate), inhibiting pathogens that produce pathogenic factors (Zafar and Saier, 2021). The intestinal flora of broilers is of paramount significance in safeguarding host health, maintaining intestinal barrier function, facilitating nutrient absorption, and enhancing production performance (Gou et al., 2019). Additionally, *Bacteroides* play a role in maintaining intestinal homeostasis, transforming bile acids, facilitating bile acid metabolism, and participating in the body’s fat synthesis (Chen et al., 2022). To further ascertain the impact of Nano-ZnO on cecal microbes, we employed LEfSe for high-dimensional analysis. Our study revealed a significant increase in the content of *anaerobicfilum* in the cecum. It has been reported that *anaerobicfilum* can produce lactate and SCFAs in the intestine, thereby enhancing intestinal immunity and feed digestibility (Wu et al., 2022). Furthermore, fecal microbial data indicated that Nano-ZnO could decrease the number of *E. coli* and *Salmonella* colonies while enriching the number of *Lactobacillus* colonies. In conclusion, Nano-ZnO may enhance the intestinal immunity and growth performance of broilers by modulating intestinal microorganisms.

## Conclusion

In summary, Nano-ZnO has a positive effect on the jejunum morphology of broilers, and is considered to be the reason for improving feed efficiency and thus growth performance. In addition, Nano-ZnO can improve serum immunoglobulin parameters of meat blood, regulate intestinal flora, and improve immunity of broilers. The data of blood biochemical indexes also proved that Nano-ZnO did not cause damage to the liver.

## Data Availability

The datasets presented in this study can be found in online repositories. The names of the repository/repositories and accession number(s) can be found below: https://figshare.com/, https://figshare.com/s/30431b58b86224fbbbfd.
